# Supplementary figures and images for: Short-term health system responses to epidemics across hard to reach areas in sub-Saharan Africa: A scoping review protocol
Source: PLoS One. 2024 Sep 18;19(9):e0285916. doi: 10.1371/journal.pone.0285916 (PMC11410208; doi:10.1371/journal.pone.0285916)

Appendix 1: Data extraction sheet


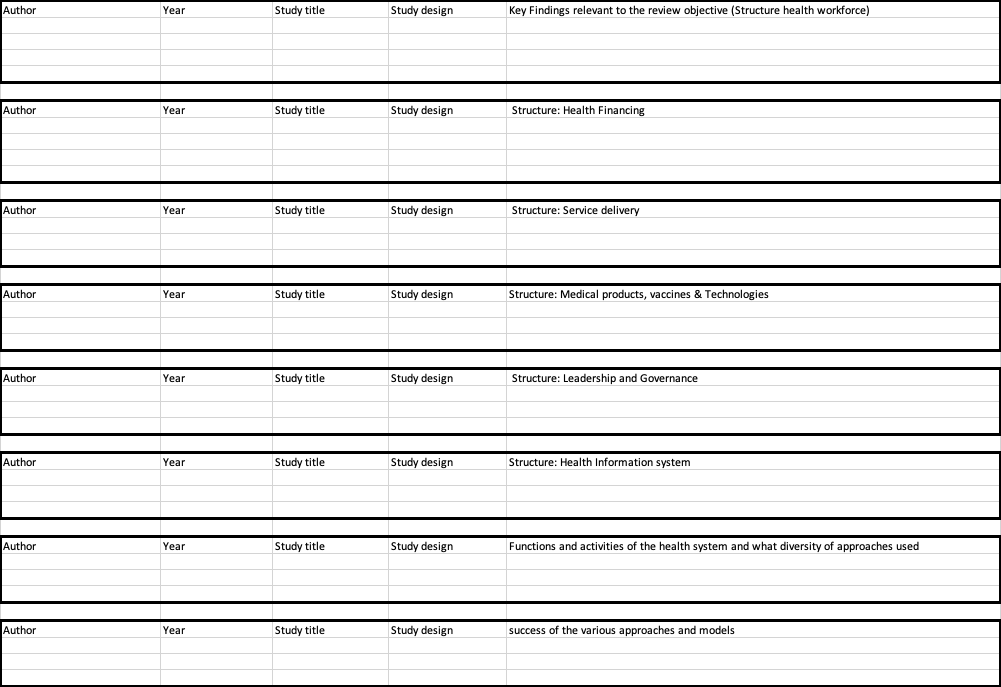

Supplement: S1 Appendix — (DOCX) [file pone.0285916.s001.docx]
