# Supplementary material for: Short-term health system responses to epidemics across hard to reach areas in sub-Saharan Africa: A scoping review protocol
Source: PLoS One. 2024 Sep 18;19(9):e0285916. doi: 10.1371/journal.pone.0285916 (PMC11410208; doi:10.1371/journal.pone.0285916)
Supplement: Attachment — Submitted filename: PONE-D-23-13244.pdf - revised.pdf [file pone.0285916.s002.pdf]

PLOS ONE

Short-term health system responses to epidemics across hard-to-reach areas in sub-Saharan Africa: A scoping review protocol

--Manuscript Draft--

**Manuscript Number:**	PONE-D-23-13244
**Article Type:**	Study Protocol
**Full Title:**	Short-term health system responses to epidemics across hard-to-reach areas in sub-Saharan Africa: A scoping review protocol
**Short Title:**	Short-term health system responses to epidemics across hard-to-reach areas in SSA: A scoping review protocol
**Corresponding Author:**	Annette Atieno Atieno, Bsc<br>SBS: Strathmore University Strathmore Business School<br>Nairobi, --- Select One --- KENYA
**Keywords:**	Epidemics; sub-Saharan region; disease outbreaks
**Abstract:**	Epidemics and disease outbreaks are the occurrence of cases of disease in excess of what would be normally expected. Epidemic-prone diseases, including emerging and re-emerging diseases, constitute the greatest threat to public health security and disruption of social and economic development. When cases of epidemics are diagnosed in specific areas, an outbreak response is triggered to stop the spread swiftly, keeping as few people as possible from being infected. In the past 20 years, the sub-Saharan region has witnessed a marked increase in the number of outbreaks in pandemics, such as cholera, dengue, A/H5N 1 influenza, and rift valley fever, among others. And while many of the efforts toward containment have been individually studied, we have no recent studies that examine them collectively in order to draw appropriate comparisons, no recent studies that have especially focused on hard-to-reach areas, and none that have applied a health systems lens. This study thus details a scoping review of short-term health system responses to epidemics across hard-to-reach areas in sub-Saharan Africa.
**Order of Authors:**	Annette Atieno Murunga, Bsc
	Ben Ngoye, PHD
	Francis Wafula, PHD
	Gilbert Kokwaro, PHD
**Additional Information:**	
**Question**	**Response**
**Financial Disclosure**<br><br>Enter a financial disclosure statement that describes the sources of funding for the work included in this submission. Review the submission guidelines for detailed requirements. View published research articles from *PLOS ONE* for specific examples.<br><br>This statement is required for submission and **will appear in the published article** if the submission is accepted. Please make sure it is accurate.	1. A. M- Wellcome (UKAid), Economic and Social Research Council, Medical Research Council- Grant Number MR/T022078/1<br><br>2. B.N- Wellcome (UKAid), Economic and Social Research Council, Medical Research Council- Grant Number MR/T022078/1<br><br>3. F.W- Wellcome (UKAid), Economic and Social Research Council, Medical Research Council- Grant Number MR/T022078/1<br><br>4. G.K- Wellcome (UKAid), Economic and Social Research Council, Medical Research Council- Grant Number MR/T022078/1<br><br>https://www.ukri.org/councils/mrc/

Powered by Editorial Manager® and ProduXion Manager® from Aries Systems Corporation

**Unfunded studies**<br>Enter: *The author(s) received no specific funding for this work.*<br><br>**Funded studies**<br>Enter a statement with the following details:<br>• Initials of the authors who received each award<br>• Grant numbers awarded to each author<br>• The full name of each funder<br>• URL of each funder website<br>• Have or will the sponsors or funders play any role in the study design, data collection (when applicable) and analysis, decision to publish, or preparation of the manuscript?<br>• **NO** - Include this sentence at the end of your statement: *The funders did not and will not have a role in study design, data collection and analysis, decision to publish, or preparation of the manuscript.*<br>• **YES** - Specify the role(s) played.<br><br>* typeset	
**Competing Interests**<br><br>Use the instructions below to enter a competing interest statement for this submission. On behalf of all authors, disclose any competing interests that could be perceived to bias this work—acknowledging all financial support and any other relevant financial or non-financial competing interests.<br><br>This statement is required for submission and **will appear in the published article** if the submission is accepted. Please make sure it is accurate and that any funding sources listed in your Funding Information later in the submission form are also declared in your Financial Disclosure statement.<br><br>View published research articles from *PLOS ONE* for specific examples.	The authors have declared that no competing interests exist.

**NO authors have competing interests**<br><br>Enter: *The authors have declared that no competing interests exist.*<br><br>**Authors with competing interests**<br><br>Enter competing interest details beginning with this statement:<br><br>*I have read the journal's policy and the authors of this manuscript have the following competing interests: [insert competing interests here]*<br><br>* typeset	
**Ethics Statement**<br><br>Enter an ethics statement for this submission. This statement is required if the study involved:<br><br>• Human participants<br>• Human specimens or tissue<br>• Vertebrate animals or cephalopods<br>• Vertebrate embryos or tissues<br>• Field research<br><br>Write "N/A" if the submission does not require an ethics statement.<br><br>General guidance is provided below. Consult the submission guidelines for detailed instructions. **Make sure that all information entered here is included in the Methods section of the manuscript.**	N/A

Powered by Editorial Manager® and ProduXion Manager® from Aries Systems Corporation

**Format for specific study types**<br><br>**Human Subject Research (involving human participants and/or tissue)**<br>• Give the name of the institutional review board or ethics committee that approved the study<br>• Include the approval number and/or a statement indicating approval of this research<br>• Indicate the form of consent obtained (written/oral) or the reason that consent was not obtained (e.g. the data were analyzed anonymously)<br><br>**Animal Research (involving vertebrate animals, embryos or tissues)**<br>• Provide the name of the Institutional Animal Care and Use Committee (IACUC) or other relevant ethics board that reviewed the study protocol, and indicate whether they approved this research or granted a formal waiver of ethical approval<br>• Include an approval number if one was obtained<br>• If the study involved *non-human primates*, add *additional details* about animal welfare and steps taken to ameliorate suffering<br>• If anesthesia, euthanasia, or any kind of animal sacrifice is part of the study, include briefly which substances and/or methods were applied<br><br>**Field Research**<br><br>Include the following details if this study involves the collection of plant, animal, or other materials from a natural setting:<br>• Field permit number<br>• Name of the institution or relevant body that granted permission	
**Data Availability**<br><br>Authors are required to make the data underlying their research findings fully available, without restriction.<br><br>If the manuscript reports pilot or preliminary results, the data underlying those results must be made available at the time of publication. A Data Availability	N/A - No results are reported

Statement describing where the data can be found is required at submission. Your answers to this question constitute the Data Availability Statement and will be published in the article, if accepted. When legal or ethical requirements prohibit public data sharing, authors must indicate how researchers may obtain access to the data. See the PLOS Data Policy and FAQ for detailed information.<br><br>**Important:** Stating 'data available on request from the author' is not sufficient. If your data are only available upon request, select 'No' for the first question and explain your situation in the text box.<br><br>For protocols without pilot or preliminary data, authors are strongly encouraged to state how they plan to share research data from their study when it is completed or published.<br><br>If your study includes pilot data, will they be made fully available without restriction?	
**Describe where the data may be found in full sentences. If you are copying our sample text, replace any instances of XXX with the appropriate details.**<br><br>• If **no pilot data are reported** in the manuscript, please enter the following: *No datasets were generated or analysed during the current study. All relevant data from this study will be made available upon study completion.*<br>• If the manuscript reports pilot data **held in a public repository**, include URLs, accession numbers or DOIs. If this information will only be available after acceptance, indicate this by ticking the box below. For example: *All XXX files are available from the XXX database (accession number(s) XXX, XXX.).*<br>• If the manuscript reports pilot data contained **within the manuscript and/or**	No datasets were generated or analysed during the current study. All relevant data from this study will be made available upon study completion.

**Supporting Information files**, enter the following:<br>*All relevant data are within the manuscript and its Supporting Information files.*<br>• If neither of these applies but you are able to provide **details of access elsewhere**, with or without limitations, please do so. For example:<br><br>*Data cannot be shared publicly because of [XXX]. Data are available from the XXX Institutional Data Access / Ethics Committee (contact via XXX) for researchers who meet the criteria for access to confidential data.*<br><br>*The data underlying the results presented in the study are available from (include the name of the third party and contact information or URL).*<br>• This text is appropriate if the data are owned by a third party and authors do not have permission to share the data.<br><br>For study protocols with data management or sharing plans, authors are encouraged to briefly describe in their Data Availability Statement how data they generate will be made accessible when the study is completed. For example:<br>• Deidentified research data will be made publicly available when the study is completed and published.<br><br>* typeset	
Additional data availability information:	

Powered by Editorial Manager® and ProduXion Manager® from Aries Systems Corporation

Manuscript Click here to access/download;Manuscript;Protocol -2.docx

Short-term health system responses to epidemics across hard-to-reach areas in sub-Saharan Africa: A scoping review protocol

Annette A. Murunga

Contributed equally to this work with: Ben Ngoye, Francis Wafula, Gilbert Kokwaro

Roles: Conceptualization, methodology, project administration, writing-original draft writing-review and editing

amurunga@strathmore.edu

Affiliation: Institute of healthcare management Strathmore Business School

Ben Ngoye

Contributed equally to this work with: Annette A. Murunga, Francis Wafula, Gilbert Kokwaro

Roles: Conceptualization, Funding acquisition, methodology, writing-review and editing

Affiliation: Institute of healthcare management Strathmore Business School

bngoye@strathmore.edu

Francis P. Wafula

Contributed equally to this work with: Annette A. Murunga, Ben Ngoye, Gilbert Kokwaro

Roles: Conceptualization, Funding acquisition, methodology, writing-review and editing

Affiliation: Institute of healthcare management Strathmore Business School

fwafula@strathmore.edu

Gilbert O. Kokwaro

Contributed equally to this work with: Annette A. Murunga, Ben Ngoye, Francis Wafula

Roles: Conceptualization, Funding acquisition, methodology, writing-review and editing

Affiliation: Institute of healthcare management Strathmore Business School

gkokwaro@strathmore.edu

Funding: This work received financial support from Wellcome (UKAid), Economic and Social Research Council, Medical Research Council- MR/T022078/1

Competing interests: The authors have declared that no competing interests exist

Data availability: All relevant data from this study will be made available upon study completion.

Abstract

Background

Epidemics and disease outbreaks are the occurrence of cases of disease in excess of what would be normally expected. Epidemic-prone diseases, including emerging and re-emerging diseases, constitute the greatest threat to public health security and disruption of social and economic development. When cases of epidemics are diagnosed in specific areas, an outbreak response is triggered to stop the spread swiftly, keeping as few people as possible from being infected. In the past 20 years, the sub-Saharan region has witnessed a marked increase in the number of outbreaks in pandemics, such as cholera, dengue, A/H5N 1 influenza, and rift valley fever, among others. And while many of the efforts toward containment have been individually studied, we have no recent studies that examine them collectively in order to draw appropriate comparisons, no recent studies that have especially focused on hard-to-reach areas, and none that have applied a health systems lens. This study thus details a scoping review of short-term health system responses to epidemics across hard-to-reach areas in sub-Saharan Africa.

Methods

A scoping review will be undertaken following PRISMA guidelines for scoping reviews. A modified Donabedian framework will be used to help in understanding approaches used in the immediate health system responses to disease outbreaks. The review will focus on published and unpublished studies that report short-term health systems responses during disease outbreaks in sub-Saharan Africa. These will be gleaned from PubMed, google scholar and Cochrane electronic databases, supplemented by a Google advanced search. In addition, manual searches will be carried out through related articles and websites. Two authors will independently screen the studies and extract data. Disagreements will be resolved by discussions, and a third reviewer will decide if there is no consensus. Data will be charted, coded, and narratively synthesized.

Outcome

We anticipate developing a document that will show the different approaches health systems responded with to epidemics in the short term and that will contribute to strengthening future short-term responses to disease outbreaks by identifying best practices and innovative ideas. In addition, the study will highlight possible knowledge gaps that need to be filled with new research.

Introduction

The Covid-19 pandemic pushed the world to the brink of a social and economic meltdown, triggering questions about how well the global health community is prepared to handle major epidemics and pandemics. The world had to deal with an event that has been described as a once-in-a-generation occurrence. That said, we cannot claim full ignorance of the risk of such calamities. Health system inequities and poor resourcing of healthcare, especially across low- and middle-income settings, put the entire world at risk. One type of population group that is particularly ignored and underfunded yet carries a substantial risk of disease outbreaks is those living in hard-to-reach areas across Africa. We define hard-to-reach areas as places where it would be hardest or take the longest for someone to access basic humanitarian services (such as health clinics and hospitals). This access is further compounded by a lack of functioning transport links and infrastructure, as well as terrain difficulty (1).

To contextualize the challenge better, though, it is important to realize that health systems in LMICs are barely able to handle those in high-access areas such as urban and more affluent areas. Major gaps exist in key areas like surveillance, emergency preparedness and response, risk communication and effective control and management of points of entry (2). A WHO evaluation reported that most SSA countries are not equipped to respond to sudden shocks to health systems, yet the region often suffers isolated disease outbreaks (3). Worse still, broader dynamics continue to contribute to the emergence and re-emergence of outbreaks of new diseases and antimicrobial resistance (4). Despite the fact that outbreaks like SARS, cholera, chikungunya, dengue fever, Rift Valley Fever and Ebola provided warning signs and tested countries' ability to respond (5), Covid-19 still found most LMICs unprepared. And the poor emergency response strategy was made worse by inadequate laboratory and epidemiological surveillance systems and economic, social and political disincentives to case reporting (6).

While Covid-19 presented a particularly unique challenge for most countries, we think that we cannot fully appreciate the path-dependent challenges affecting SSA without going back in time to understand how countries have been responding to other types of outbreaks. We are particularly keen to document immediate (short-term) responses in order to better understand policy agility and capacity to learn, adapt and change when the need does not

seem/appear to be urgent. We know that because most SSA countries focus on urgent rather than important matters, their ability to handle Covid-19, which is both an urgent and important matter, was compromised. We argue that by understanding immediate policy response to outbreaks in low-priority areas, we may be able to characterize and deal with the pain points that prevent countries from prioritizing and establishing mechanisms to nip outbreaks in the bud before the major calamity. We also argue that while effort has gone towards examining health sector responses to specific outbreaks, not enough attention has gone towards characterizing commonalities in immediate response to the disease of different kinds and how the immediate actions translate into (or fail to translate into) medium- and longer-term strategies. Examining the causal relationships between the response activities and the broader health system change is crucial if the global health community is to find a lasting solution to disease outbreaks. Moreover, successful strategies for pandemic response and preparedness over the years have been viewed from a vertical lens that is disease-specific, yet it is plausible that if the response was looked at from a health system lens, the response would be quick and more effective. This explicit linkage appears under-addressed by existing literature.

Our scoping review, therefore, aims to provide an overview of existing evidence on immediate health system responses to epidemics in SSA and answer the following questions: what is the context of the health system (Service Delivery, Human Resources, Leadership and Governance, Financing, Medicines Procurement and Supply Chain, and Information Technology) with regard to epidemic preparedness and management in SSA? What is the diversity of approaches in terms of policies, protocols, communication, community participation, and surveillance that were used to manage disease outbreaks? And what were the outcomes of the various approaches and models in the early detection of diseases, reduced disease impact on the population, change in health behaviour, and strengthened health system?

The rationale for the scoping review

A scoping review is suitable for summarizing and disseminating research findings and identifying research gaps in the existing literature. While a systematic review typically focuses

on a well-defined question where appropriate study designs can be identified in advance, a scoping study addresses broader topics where different study designs may be applicable (7).

Conceptual framework for the scoping review

A conceptual framework has been developed using the Donabedian framework (8) of structure, processes and outcomes (Figure 1). The framework is commonly used to assess the quality of health services. For this review, a modified framework will be used to help in understanding approaches used in the immediate health system responses to disease outbreaks. For the review, ***Structures*** will describe the context of the health system and includes the WHO building blocks of Service Delivery, Human Resource for health (HRH) , Leadership Management and Governance (LMG), Financing, Medicines, and Technologies, Health Information Systems (HMIS) (9). ***The process*** will include the functions and the surveillance, policies, protocols, and communication between partners, and ***outcomes*** will include early detection, reduced disease impact on the population and improved health systems.

Aided by this modified framework, we focus on the following review questions:

1. Context of the health system building block: *What contextual forces are shaping the health system, and what are the key challenges faced by countries in SSA?*
2. Processes (functions and activities) of the health system: *What are the main functions and activities of the health system in SSA, and what diversity of approaches, models, innovations and tools were used to manage disease outbreaks?*
3. Outcomes of the health system: *How successful are the various approaches and models in improving the prevention and management of disease outbreaks?*

Methodology

The review seeks to describe the short-term health system responses to epidemics across hard-to-reach areas in sub-Saharan Africa. Like other scoping reviews, the purpose is to broadly describe a phenomenon (what is known and what isn't) and characterize it in ways that contribute towards a more detailed understanding and/or further research. Consequently, we seek to identify and map available evidence from a wide variety of sources,

including published and unpublished material. We follow Arksey and O'Malley's five-stage scoping review framework (10), which proposes the following five stages: identifying the research question, finding the articles, selecting appropriate articles, charting the data, and collating and summarizing the data into meaningful themes.

Stage 1: Identifying the research question

The research question in this review is based on the Joanna Briggs Institute PCC (Population-concept-context) model (11). An overarching research question was developed to guide the search strategy: “What are the short-term health system responses to epidemics across hard-to-reach areas in sub-Saharan Africa?”. The question allows us to capture appropriate existing literature and also provides an opportunity for research questions to be added or modified in the course of the study.

Stage 2: Identifying relevant studies

Our approach will include a systematic search of peer-reviewed studies from electronic databases - PubMed, google scholar and Cochrane, snowballing from article reference lists to include additional studies that may not have been indexed. We will also search grey literature from the internet using Google. Literature search strategies will be developed using medical subject headings (MeSH-terms) and text words related to the population, geographical terms and phenomenon of interest/intervention, as shown in Table 1.

Empirical studies with either qualitative or quantitative data published in English will be considered for inclusion. The scoping review will exclude all types of reviews, protocols and book chapters. A three-step search strategy will be undertaken. An initial limited search of PubMed, google scholar and Cochrane will be carried out, followed by an analysis of the text words contained in the title and abstract and the index terms used. The second step will include using all identified keywords and index terms in all the included databases. The third step will include screening the reference list of all the studies for additional arti[illegible]. We shall consider all articles from 2010 onwards.

MeSH terms used to search for CAB Direct included: *Epidemic OR disease outbreak, AND Health system response AND Sub-Saharan Africa*; and for Google Scholar, *Health system response to epidemics AND Sub-Saharan Africa*

Table 1: Search terms used in searching Pub-Med electronic database

Group A: Target population terms (combined by 'OR')	Group B: Geographical terms (combined by 'OR')	Group C: intervention (combined by 'OR')
AND cholera OR diarrhoea OR diarrhoea OR dysentery OR Covid-19 OR leishmaniasis OR kala azar OR trypanosomiasis OR sleeping sickness OR malaria OR polio OR measles OR mumps OR meningitis OR chikungunya OR yellow fever OR dengue OR Ebola OR SARS OR MERS OR influenza OR Zika OR epidemic	AND Angola OR Benin OR Burkina Faso OR Burundi OR Cape Verde OR Central African Republic OR Chad OR Comoros OR the Democratic Republic of Congo OR Djibouti OR Equatorial Guinea OR Eritrea OR Ethiopia OR Gambia OR Guinea OR Guinea-Bissau OR Lesotho OR Liberia OR Madagascar OR Malawi OR Mali OR Mauritania OR Mozambique OR Niger OR Rwanda OR Sao Tome and Principe OR Senegal OR Sierra Leone OR Somalia OR Sudan OR Tanzania OR Togo OR Uganda OR Zambia OR Cameroon OR Congo OR Côte d'Ivoire OR Ivory Coast OR Ghana OR Kenya OR Nigeria OR Zimbabwe OR Namibia OR Swaziland OR Botswana OR Gabon OR Mauritius OR South Africa OR Seychelles OR Sub-Saharan Africa OR hard-to-	Health systems impact OR Health systems resilience OR Health systems performance OR Health systems response OR Health system assessment, OR Immediate health system response, OR Short-term health system response

reach areas in Sub-Saharan Africa

OR ASAL areas in SSA

Stage 3: Selecting articles

Following the search, all identified articles will be uploaded onto Zotero, where all duplicates will be removed. The review process will be carried out in two stages; each stage will have two reviewers who will independently determine the articles based on the inclusion and exclusion criteria. The first screening process will include reading the title and the abstracts to reach the following decisions: (1) if at least one reviewer agrees to include or consider the abstract or title to be inconclusive, the study will be moved to the next level of screening; (2) for any of the studies if both reviewers agree for an article to be excluded, the article will be excluded. All studies at this level will be charted in an Excel sheet.

In the second level of screening, the full texts will be assessed by two independent reviewers. Reasons for any exclusion will be recorded and reported in the scoping review. Any disagreements will be resolved through discussions or with a third reviewer. The results of the search and study selection will be reported in a Preferred Reporting Items for Systematic Reviews and Meta-Analyses (PRISMA) flow diagram.

Stage 4: Charting data

To answer the research question, a data charting form in Excel with the following features will be created: authors, year of publication, study title, study design, the aim of the study, country of the study, and key findings relevant to the review objective. A draft charting table is provided (appendix 1). As a preliminary step, the reviewers will independently extract data from the first five articles using the data-charting table and meet to determine whether the approach to data extraction is consistent with the purpose of the study. The draft data extraction tool will be modified and revised as necessary during the extraction process. Modifications will be detailed in the full scoping review article.

Stage 5: collating, summarizing and reporting the results

The data from stage 4 will be collated, summarized and reported in a manner that aligns with the study objectives. Tabular and graphical representations of the data may be used to illustrate the identified results and will be supported by narrative descriptions of the data.

The meaning of the findings and implications for future research and practice will be discussed.

Patient and public involvement

Patients and the public will not be involved in the design and conception of the study.

Ethics and dissemination

The proposed scoping review does not require ethical approval as data will be collected through published peer-reviewed literature and grey literature. This scoping review will provide a comprehensive overview of existing evidence in the field.

References

1. C-03-WDR-2018-3-reach.pdf [Internet]. [cited 2021 Jul 22]. Available from: https://media.ifrc.org/ifrc/wp-content/uploads/2018/10/C-03-WDR-2018-3-reach.pdf

2. Lokossou VK, Usman AB, Sombie I, Paraiso MN, Balogun MS, Umeokonkwo CD, et al. COVID-19 pandemic in Economic Community of West African States (ECOWAS) region: implication for capacity strengthening at Point of Entry. Pan African Medical Journal [Internet]. 2021 May 25 [cited 2023 Apr 18];39(1). Available from: https://www.ajol.info/index.php/pamj/article/view/219655

3. Paintsil E. COVID-19 threatens health systems in sub-Saharan Africa: the eye of the crocodile. J Clin Invest [Internet]. 2020 Jun 1 [cited 2023 Apr 18];130(6):2741–4. Available from: https://www.jci.org/articles/view/138493

4. Cohen ML. Changing patterns of infectious disease. Nature. 2000 Aug;406(6797):762–7.

5. Cable J, Heymann DL, Uzicanin A, Tomori O, Marinissen MJ, Katz R, et al. Pandemic diseases preparedness and response in the age of COVID-19-a symposium report. Ann N Y Acad Sci. 2021 Apr;1489(1):17–29.

6. Ali M, Lopez AL, Ae You Y, Eun Kim Y, Sah B, Maskery B, et al. The global burden of cholera. Bull World Health Org. 2012 Mar 1;90(3):209–18.

7. Haugdahl HS, Sandsæter HL, Lysne M, Bjerkeset O, Uhrenfeldt L, Horn J. Postpartum lifestyle interventions among women with pre-eclampsia: a scoping review protocol. BMJ Open [Internet]. 2020 May 1 [cited 2021 Jul 22];10(5):e035507. Available from: https://bmjopen.bmj.com/content/10/5/e035507

8. Voyce J, Gouveia M, Medinas M, Santos A, Ferreira R. A Donabedian Model of the Quality of Nursing Care From Nurses' Perspectives in a Portuguese Hospital: A Pilot Study. Journal of Nursing Measurement. 2015 Dec 1;23:474–84.

9. World Health Organization, editor. Monitoring the building blocks of health systems: a handbook of indicators and their measurement strategies. Geneva: World Health Organization; 2010. 92 p.

10. Arksey H, O'Malley L. Scoping studies: towards a methodological framework. 2007 Feb [cited 2021 Jun 24];8. Available from: https://www.tandfonline.com/doi/abs/10.1080/1364557032000119616

11. Peters M, Godfrey C, Khalil H, Mcinerney P, Soares C, Parker D. 2017 Guidance for the Conduct of JBI Scoping Reviews. In 2017.

Click here to access/download;Figure;Data extraction sheet.docx

Appendix 1: Data extraction sheet

Author	Year	Study title	Study design	Key Findings relevant to the review objective (Structure health workforce)

Author	Year	Study title	Study design	Structure: Health Financing

Author	Year	Study title	Study design	Structure: Service delivery

Author	Year	Study title	Study design	Structure: Medical products, vaccines & Technologies

Author	Year	Study title	Study design	Structure: Leadership and Governance

Author	Year	Study title	Study design	Structure: Health Information system

Author	Year	Study title	Study design	Functions and activities of the health system and what diversity of approaches used

Author	Year	Study title	Study design	success of the various approaches and models

Click here to access/download;Figure;Donabedian framework for the scoping review.docx

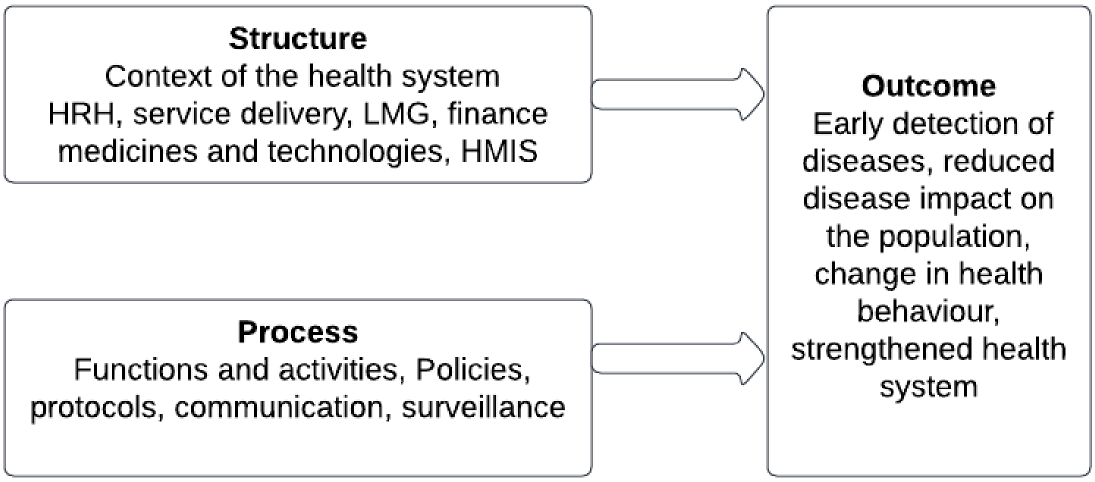

Figure 1: Modified Donabedian framework for the scoping review

Prisma flow diagram
Click here to access/download;Figure;PRISMA flow diagram.docx

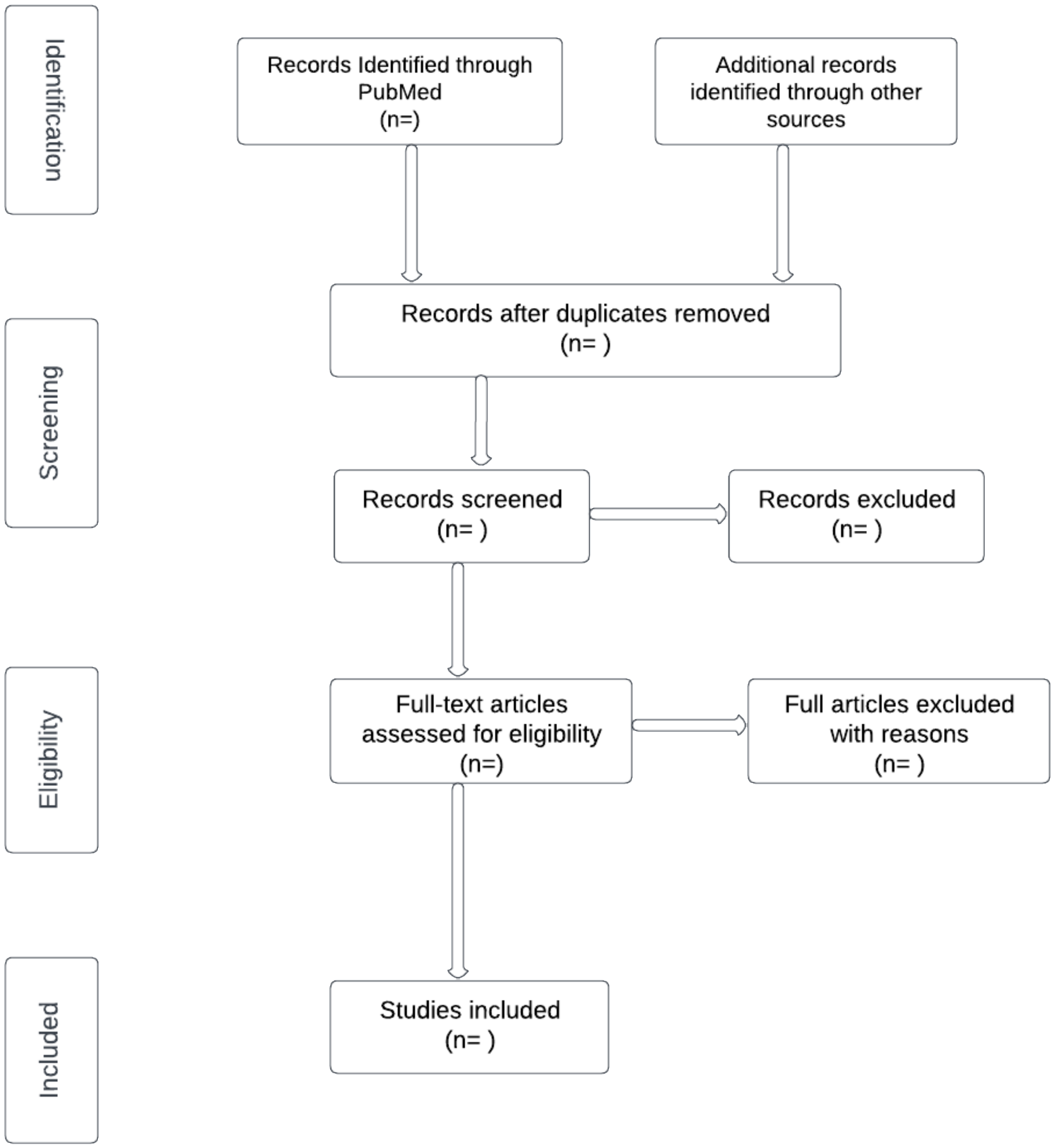

Figure 2; Preferred Reporting Items for Systematic Reviews and Meta-Analyses (PRISMA) flow diagram.
